# A lived experience co-designed study protocol for a randomised control trial: the Attempted Suicide Short Intervention Program (ASSIP) or Brief Cognitive Behavioural Therapy as additional interventions after a suicide attempt compared to a standard Suicide Prevention Pathway (SPP)

**DOI:** 10.1186/s13063-021-05658-y

**Published:** 2021-10-21

**Authors:** Nicolas J. C. Stapelberg, Candice Bowman, Sabine Woerwag-Mehta, Sarah Walker, Angela Davies, Ian Hughes, Konrad Michel, Anthony R. Pisani, Heidy Van Engelen, Mia Delos, Tamara Hageman, Kim Fullerton-Smith, Ravikumar Krishnaiah, Sarah McDowell, Alison Cameron, Trudy-Lee Scales, Cherie Dillon, Titta Gigante, Cindy Heddle, Natalie Mudge, Anne Zappa, Michelle Edwards, Sigi Gutjahr, Hitesh Joshi, Kathryn Turner

**Affiliations:** 1 Department of Mental Health and Specialist Services Gold Coast University Hospital, 1 Hospital Boulevard, Southport, Queensland 4215 Australia; 2 Faculty Health Sciences and Medicine, Bond University, 14 University Drive, Robina, Queensland 4226 Australia; 3 Lived Experience Suicide Prevention Research Advisory Committee (this committee was convened specifically for this study and is supported by the Gold Coast Mental Health and Specialist Services Peer Workers), Southport, Australia; 4 Office for Research Governance and Development, Gold Coast Health, 1 Hospital Boulevard, Southport, Queensland 4215 Australia; 5 University Hospital of Psychiatry and Psychotherapy, University of Bern, Bern, Switzerland; 6 Departments of Psychiatry and Pediatrics, University of Rochester, 300 Crittenden Blvd., BOX PSYCH, Rochester, NY 14642 USA

**Keywords:** Suicide prevention, Cognitive behavioural therapy (CBT), Attempted Suicide Short Intervention Program (ASSIP), Self-harm, Suicidality, Suicide attempt, Lived experience, Emergency department, Mental health, Brief intervention

## Abstract

**Background:**

Despite being preventable, suicide is a leading cause of death and a major global public health problem. For every death by suicide, many more suicide attempts are undertaken, and this presents as a critical risk factor for suicide. Currently, there are limited treatment options with limited underpinning research for those who present to emergency departments with suicidal behaviour. The aim of this study is to assess if adding one of two structured suicide-specific psychological interventions (Attempted Suicide Short Intervention Program [ASSIP] or Brief Cognitive Behavioural Therapy [CBT] for Suicide Prevention) to a standardised clinical care approach (Suicide Prevention Pathway [SPP]) improves the outcomes for consumers presenting to a Mental Health Service with a suicide attempt.

**Methods:**

This is a randomised controlled trial with blinding of those assessing the outcomes. People who attempt suicide or experience suicidality after a suicide attempt, present to the Gold Coast Mental Health and Specialist Services, are placed on the Suicide Prevention Pathway (SPP), and meet the eligibility criteria, are offered the opportunity to participate. A total of 411 participants will be recruited for the study, with 137 allocated to each cohort (participants are randomised to SPP, ASSIP + SPP, or CBT + SPP). The primary outcomes of this study are re-presentation to hospitals with suicide attempts. Presentations with suicidal ideation will also be examined (in a descriptive analysis) to ascertain whether a rise in suicidal ideation is commensurate with a fall in suicide attempts (which might indicate an increase in help-seeking behaviours). Death by suicide rates will also be examined to ensure that representations with a suicide attempt are not due to participants dying, but due to a potential improvement in mental health. For participants without a subsequent suicide attempt, the total number of days from enrolment to the last assessment (24 months) will be calculated. Self-reported levels of suicidality, depression, anxiety, stress, resilience, problem-solving skills, and self- and therapist-reported level of therapeutic engagement are also being examined. Psychometric data are collected at baseline, end of interventions, and 6,12, and 24 months.

**Discussion:**

This project will move both ASSIP and Brief CBT from efficacy to effectiveness research, with clear aims of assessing the addition of two structured psychological interventions to treatment as usual, providing a cost-benefit analysis of the interventions, thus delivering outcomes providing a clear pathway for rapid translation of successful interventions.

**Trials registration:**

ClinicalTrials.govNCT04072666. Registered on 28 August 2019

## Administrative information

Note: the numbers in curly brackets in this protocol refer to SPIRIT checklist item numbers. The order of the items has been modified to group similar items (see http://www.equator-network.org/reporting-guidelines/spirit-2013-statement-defining-standard-protocol-items-for-clinical-trials/).

**Table:** 

Title {1}	A Lived Experience co-designed study protocol for a randomised control trial: The Attempted Suicide Short Intervention Program (ASSIP) or Brief Cognitive Behavioural Therapy as additional interventions after a suicide attempt compared to a standard Suicide Prevention Pathway (SPP).
Trial registration {2a and 2b}.	NCT04072666 - Registered on 28^th^ August 2019 on Clinical Trials US Gov (clinicaltrials.gov) and ANZCTR: https://www.clinicaltrials.gov/ct2/show/NCT04072666?term=NCT04072666&draw=2&rank=1 https://www.anzctr.org.au/TrialSearch.aspx
Protocol version {2}	Protocol version 1.0, 5th April 2019.
Funding {2}	Suicide Prevention Australia is funding the costs for this trial up to 411 participants recruited. Bond University is providing additional funding for a higher degree research stipend and for additional research assistant support. Gold Coast Mental Health and Specialist Services cover organisational costs and in-kind staff support.
Author details {5a}	1. Division of Mental Health and Specialist Services Gold Coast University Hospital, 1 Hospital Boulevard, Southport, Queensland, 4215 Australia. 2. Office for Research Governance and Development, Gold Coast Health, 1 Hospital Boulevard, Southport, Queensland, 4215 Australia. 3. Lived Experience Suicide Prevention Research Advisory Committee (this committee was convened specifically for this study and is supported by the Gold Coast Mental Health and Specialist Services peer workers). 4. Faculty Health Sciences and Medicine, Bond University, 14 University Drive, Robina, Queensland, 4226, Australia. 5. Department of Psychology, University of Rochester, New York State, 601 Elmwood Ave, box PSYCH Rochester, NY 14642, USA 6. University Hospital of Psychiatry and Psychotherapy, University of Bern, Switzerland
Name and contact information for the trial sponsor {5b}	Clinical Director Gold Coast Mental Health and Specialist Services: Dr Kathryn Turner Address: Ground Floor, Block F, Gold Coast University Hospital (GCUH) 4215 Telephone: 07 5687 7249 Email: kathryn.turner@health.qld.gov.au
Role of sponsor and funder {5c}	The funding bodies (Suicide Prevention Australia and Bond University) will have no role in the study design; collection, management, analysis, and interpretation of data, and will not be involved in the writing of the manuscript.

## Introduction

### Background and rationale {6a}

Every year, across the globe, approximately 800,000 people die by suicide, reflecting an age-standardised annual rate of 10.6 deaths per 100,000 people [1]. In Australia, 3128 people died by suicide in 2017, increasing from 2866 in 2016 [2]. In 2017, suicide was the leading cause of death for all persons aged 15 to 44 years, with the median age for suicide being 44.5 years (an increase from 43.3 years in 2016). Suicide accounted for 36% of deaths among people aged 15–24 years and 30.9% of deaths among those aged 24–34 years [2]. Males die by suicide at a rate three times that of females [2]. Suicide deaths account for a greater proportion of deaths among Indigenous Australians (5.5%) when compared with non-Indigenous Australians (2.0%) [2]. Other groups considered to be at increased risk of suicide include people living in rural and remote areas [3]; people with disabilities [4]; people with mental illness [5]; people who identify as lesbian, gay, bisexual, transgender, intersex, and queer (LGBTIQ) [6]; and immigrants [7, 8].

For every death by suicide, many more suicide attempts are undertaken; this presents as a critical risk factor for suicide [9]. An Australian community survey study estimated that 23 suicide attempts are made for every suicide [10]. Likewise, the World Health Organization estimated that approximately four out of every 1000 adults have made a suicide attempt [9]. Local data from the Gold Coast University Hospital Emergency Department showed that from 2009 to 2018, presentations in suicide crisis showed an increasing trend. In particular, the rate of suicidal presentations (including suicide ideation and suicide attempts) was six times higher than population growth in this period [11]. This is significant in light of suicide attempts and intentional self-harm being recognised as the strongest predictors of future suicidal behaviour [9].

Current national suicide prevention programmes have highlighted the knowledge that suicide is a behaviour that stems from a complex and multifaceted set of circumstances and individual characteristics (e.g. 12, 13). These factors can be present across the human lifespan and occur across multiple cultural and community settings [12]. The complex, heterogeneous nature of the factors influencing suicide rates requires a collaborative and coordinated systems approach to prevention, incorporating strategies simultaneously implemented across multiple levels, including service systems, individualised interventions, and community prevention [14]. The efficacy of various suicide prevention interventions has been the subject of research for some time and includes a number of recent systematic reviews (e.g. [14–16]).

For instance, a recent 10-year systematic review on suicide prevention strategies showed that restricting access to lethal means (especially controlling analgesics and popular suicide jumping sites) reduces death by suicide [14]. Following up individuals who have made a suicide attempt (e.g. by phone or by letter) can also reduce suicide-related behaviours [14]. Pharmacological and psychological treatment for depression can reduce suicide thinking and behaviour in adults, children, and adolescents [14]. Psychotherapies, such as CBT and dialectical behaviour therapy (DBT), have been found to be effective in reducing suicide ideation and behaviour in adults and adolescents compared to treatment as usual (TAU) [12].

Some have argued that psychotherapy (or talking therapy) should be at the centre of suicide prevention, as suicide is a goal-directed action [17] and individuals make a decision to end their life [17, 18]. The Lancet Psychiatry Commission on psychological treatments argues that interventions should focus on the specific psychological aspects associated with suicidal behaviour, such as “feelings of defeat, entrapment, not belonging, and being a burden, as well as future thinking, goal adjustment, reasons for living, and fearlessness of death” ([18], p. 271). People who suffer from a range of psychiatric disorders (e.g. depression, anxiety, personality disorders, eating disorders) also seem to prefer psychological therapy over pharmacological treatment [19]. Moreover, psychological research aimed at developing brief interventions targeting suicidal ideation and behaviour can potentially deliver treatment options more quickly than drug-related research for mental disorders [18].

Despite the importance of psychological treatments for mental health, evidence-based interventions aimed at improving resilience and coping skills are lacking for people who present with suicidal ideation and suicide attempts to emergency departments (EDs) in Australia [18]. Effective psychological interventions could also provide an improvement in depression, anxiety, and stress symptoms; coping strategies and resilience; and a reduction in representation rates to emergency settings with attempted and completed suicides. Furthermore, there is a need to deliver new and innovative psychological interventions that are based on the most updated models and research [18].

Finally, there is a growing consensus that people with lived experience of suicide should be involved in all aspects of suicide prevention, including research into effective treatments [18].

#### Lived experience

To date, individuals with lived experience of mental health have generally been involved in service development and service evaluation [20]. There is now a growing movement to involve individuals with lived experience in research, not only in consultation, but in co-design of research studies and as active co-investigators (for a review, see [20]). Meaningful involvement of individuals with lived experience will contribute to better outcomes in research and in mental health care [21]. Moreover, issues that are important to individuals with lived experience (both consumers and their carers) can be identified and prioritised through research involvement [22]. In addition, individuals with lived experience are able to provide unique insights into all aspects of the research (e.g. study design, recruitment, interpretation of the findings, and dissemination); for example, individuals with lived experience can explore the potential adverse effects of a particular psychological therapy as well as provide insight into how the therapy is likely to be received by potential participants [18]. Individuals with lived experience can also aid in improving the retention of research participants in a therapy trial [18].

A Lived Experience Suicide Prevention Research Advisory Committee has been specifically convened for this study. There are seven members of the Lived Experience Suicide Prevention Research Advisory Committee. In addition, the Committee is supported by Gold Coast Mental Health and Specialist Services (GCMHSS) peer workers and is led by our GCMHSS Consumer Representative and our GCMHSS Carer Representative. One member of the Committee identifies as an emerging Aboriginal elder, who also has lived experience of suicidality. There are also culturally and linguistically diverse (CALD) representative people on the Committee. Under the guidance of our Gold Coast Health Consumer Representative and our Gold Coast Health Carer Representative, there has already, and will continue to be, substantial interface with the lived experience and consumer community. This will aid to gauge the participants’ and communities’ expectations of the research, and we are being guided by the Committee to manage any relevant limitations of the research project.

### Objectives {7}

The aim of this study is to assess if adding one of two manualised suicide specific psychological interventions to a standardised clinical care approach improves primary and secondary clinical outcomes for consumers presenting to a Mental Health Service with a suicide attempt.

1. Attempted Suicide Short Intervention Program (ASSIP) is a novel, manualised therapy, composed of three therapy sessions following a suicide attempt, with subsequent follow up over 2 years with personalised mailed letters [23].
2. Brief Cognitive Behavioural Therapy (CBT) for Suicide Prevention is a manualised approach involving brief CBT for suicide in six 60-min sessions. The intervention incorporates skills development and emphasises internal self-management [24, 25].

The standard care approach involves a Suicide Prevention Pathway (SPP) modelled on the Zero Suicide Framework [26]. It utilises a comprehensive assessment, the chronological assessment of suicide events (CASE) approach to elicit suicidal intent [27], Pisani’s Prevention Orientated Risk Formulation [28], Safety Planning (including counselling on access to lethal means) [29, 30], consumer and carer education, individualised care planning, rapid referral, structured follow-up [31], and safe transitions of care.

To address the central aim, we are comparing primary and secondary clinical outcomes for the following:

1. Three sessions of the Attempted Suicide Short Intervention Program (ASSIP) + SPP, versus SPP alone
2. Six sessions of Brief Cognitive Behavioural Therapy (CBT) + SPP, versus SPP alone

Suicide places a substantial economic cost on both health services and the community. As such, the service will also undertake a cost comparison to examine the relative costs of each of the three intervention pathways being examined in this study. Service-related information is regularly collated by the GCMHSS and ED (i.e. contact hours, bed stays, ED visits), and costs will be estimated based on this information.

#### Hypotheses

1. The use of suicide-specific psychological interventions (ASSIP; CBT) combined with a comprehensive clinical SPP will have better outcomes than the clinical SPP alone.
2. Outcomes for the ASSIP + SPP and CBT + SPP will be similar. This hypothesis is exploratory in nature.

### Trial design {32}

This is a randomised controlled trial, with blinding of those assessing the outcomes. There are three arms: intervention group (ASSIP+SPP or CBT + SPP) and the treatment as usual group (SPP). Randomisation occurs after recruitment with 1:1:1 allocation.

After consenting, consumers are allocated randomly to one of the following three groups:

1. Suicide Prevention Pathway (SPP, standard care approach)
2. Attempted Suicide Short Intervention Program (ASSIP)
3. Brief Cognitive Behavioural Therapy (CBT) for Suicide Prevention

The study protocol for this study has been approved by the Gold Coast Health Human Research and Ethics Committee (HREC) (Application Approval number HREC/2019/QGC/51361).

## Methods: participants, interventions, and outcomes

### Study setting {9}

All aspects of the study are taking place at the Gold Coast Mental Health and Specialist Services (GCMHSS) in Queensland, Australia. The Gold Coast region has a population of around 593,200 and is projected to grow at a rate of 27% by 2026. Gold Coast Health delivers a broad range of secondary and tertiary health services across two public hospitals and several health precincts and community health centres. Databases and processes used for the evaluation of the SPP clinical pathway provide an established infrastructure for undertaking research to further the understanding of the clinical needs of this population. Research arising from the SPP will be directly translated into clinical services to optimise addressing the needs of the Gold Coast population. The GCMHSS is well placed to access large volumes of rigorous data, to provide evidence-based insight into the implementation challenges of a large-scale clinical suicide prevention strategy, and to provide information on the characteristics of people in crisis with suicidal presentations and their acute psychiatric management.

### Eligibility criteria {10}

People aged 16 and above who attempt suicide and present to the GCMHSS, who are placed on the SPP and who meet the study eligibility criteria, are offered the opportunity to participate. All participants undergo a formal informed consent process by the research assistant (RA) and a research clinician. The interventions are delivered by the research clinicians.

#### Inclusion criteria

Inclusion criteria for people going on the clinical SPP pathway are as follows: presenting to the GCMHSS with a suicide attempt, presenting with suicidal ideation and having a past history of a suicide attempt, or presenting with a suicidal presentation (suicide attempt or ideation or non-suicidal self-injury [NSSI]) and being deemed eligible for the SPP as per a psychiatrist’s clinical assessment.

The inclusion criteria for the study were people on the SPP, who had made a suicide attempt. People eligible to participate in the study were thus a subset of people treated on the SPP.

#### Exclusion criteria

The following are the exclusion criteria: decline of, or inability to, consent; inability to consent may include a significant language or speech problems, acute psychosis/thought disorder, cognitive impairment, or significant developmental disorder (e.g. learning disabilities, autism, dementia); and people with a currently clinically relevant diagnosis of borderline personality disorder are excluded, as ASSIP is not recommended for consumers with a history of complex trauma (Michel 2018, developer of ASSIP); however, clinicians can refer at their own discretion following an MDT discussion.

People who are already receiving specialised psychological interventions or who are taking psychotropic medication will still be able to participate in this study. We follow a pragmatic study design model [32], with the study being conducted in a busy “real-world” mental health setting to test the applicability of these interventions to consumers from different backgrounds, including previous service use and treatment plans. Information about dropout for each individual (including reasons for dropping out) is being collected for this study and defined as a participant who completes the baseline assessment (conducted at the same time as the formal consent process), but does not attend any of the follow-up assessment or treatment sessions since that. If a person has been rebooked three times or more and does not attend, they will be considered as someone who has dropped out of the study; that person is then discussed in MDT (e.g. for referral back to the Acute Care Team if appropriate). A CONSORT diagram will be provided in the publication of results [33].

### Who will take informed consent? {26a}

The Lived Experience Suicide Prevention Research Advisory Committee has provided specific feedback on the content and language of the information and consent forms, when and how to approach potential participants, and time frames for recruitment relative to likely distress. It was agreed that approaching potential participants with information about the study was reasonable between 48- and 72-h post-event.

Participants are approached with information about the study by the clinician or a trained research assistant (RA) between 48- and 72-h post-event. Participants are allowed time for consideration of their participation, with RAs following up if more time is required.

The RAs and clinicians then obtain formal consent.

### Additional consent provisions for collection and use of participant data and biological specimens {26b}

This is not applicable, as no additional participant data or biological specimens will be used in ancillary studies.

### Interventions

#### Explanation for the choice of comparators {6b}

Both ASSIP and the Brief CBT for Suicide Prevention have an existing evidence base and both have been shown to be cost-effective in their own right [17, 34]. In addition, the effect and cost-effectiveness of both interventions are not known when delivered in the context of a clinical Suicide Prevention Pathway, which has, itself, been shown to reduce representations with suicide attempts compared to traditional treatment [35]. Different interventions may work for different people (e.g. video-assisted narrative vs. traditional approach to identifying core beliefs driving behaviour), so it is important for the field to know which works best with whom.

ASSIP is an evidence-based intervention for reducing suicidal behaviour, while also reducing healthcare costs [36]. In the seminal randomised controlled trial (RCT) of ASSIP, conducted in Switzerland by the developers of the intervention, the ASSIP treatment, which added one session of risk assessment to treatment as usual, reduced suicide reattempts dramatically compared to treatment as usual. Over a 24-month period, individuals in the treatment group had an 80% risk reduction for suicide reattempt and an average of 72% fewer hospital days than control patients over 24 months [17, 37, 38].

CBT can challenge maladaptive beliefs, improve problem-solving skills, and social competence. Systematic reviews and meta-analyses have found CBT to be highly effective in reducing suicidal behaviour [34]. Denchev et al. found CBT to be a cost-effective intervention that reduced suicide risk among patients who presented to general hospital EDs [39]. A systematic review of the evidence on CBT concluded that it was effective in reducing self-harm behaviour and repeated suicide attempts in fewer than 10 individual sessions in patients who had made a previous suicide attempt [40]. Brief CBT for suicide prevention compared to conventional CBT has the advantage of being more cost-effective and time-efficient [24].

The standard care approach involves a Suicide Prevention Pathway (SPP) modelled on the Zero Suicide Framework [26]. It utilises a comprehensive assessment, the CASE approach to elicit suicidal intent [27] and a Prevention Orientated Risk Formulation model (i.e. risk formulation that takes into account a person’s risk status, risk state, available resources, and foreseeable changes) [28]. This is followed by safety planning (including counselling on access to lethal means) with the consumer during their initial assessment and prior to the above outlined additional treatment interventions [29, 30], brief patient/carer education, individualised care planning, rapid referral, structured follow up [31], and safe transitions of care to further providers.

#### Intervention description {11a}

##### Attempted Suicide Short Intervention Program (ASSIP)

ASSIP is a manualised brief therapy composed of three therapy sessions following a suicide attempt and subsequent follow-up over 2 years with personalised mailed letters [23]. The first session consists entirely of a video-recorded, narrative interview with the consumer relating the personal story of how the point of attempting suicide was reached. The second session involves the therapist and consumer watching the video of the recorded session together and collaboratively reflecting on the suicidal dynamic in a safe environment. Automatic thoughts, emotions, psychological pain and stress, and contingent behaviour are discussed. A psycho-educative handout and homework are given to consumers. The third session starts with a discussion of the homework. This is followed by jointly formulating the ASSIP case conceptualisation. A credit card size leaflet (i.e. a leporello) is provided on which is printed the agreed long-term goals, individual warning signs, and safety strategies. A second card is provided with crisis contact phone numbers. These sessions are followed by letters, which asks consumers how things were going [38]. These letters (written by the therapist) are sent regularly over 24 months (i.e. every 3 months in the first year, and every 6 months in the second year) [38].

##### Brief Cognitive Behavioural Therapy (CBT) for Suicide Prevention

This is a manualised approach involving brief CBT for suicide in six 60-min sessions and has been adapted by the research team. After a comprehensive search of the literature, various online CBT resources were accessed to create the manual. More specifically, permission was obtained from two authors who had created and published CBT manuals for the treatment of suicide: the Pakistan Institute of Living and Learning [41] and Stewart [42]. The elements of both these manuals were taken to create the Brief CBT for Suicide Prevention manual used for this study. The Brief CBT for Suicide Prevention incorporates skills development and emphasises internal self-management [24]. The therapy focuses on the identification of internal, external, and/or thematic triggers for suicidal thinking and behaviours, as well as factors that maintain the desire to suicide, using thought records and/or chain analyses. The brief CBT for Suicide Prevention aims to challenge distortions and misconceptions, including core beliefs that interfere with the motivation to initiate the process of problem-solving and distress tolerance, by working on acceptance of emotional and/or physical pain. The final phase of the brief intervention focuses on relapse prevention [43].

##### Suicide Prevention Pathway (standard care approach)

The SPP comprises seven inter-related clinical steps that are undertaken with consumers presenting with suicidality:

i) Initial screening—persons experiencing suicide ideation and who may also have a history of, or recent, suicide attempt, are placed on the pathway.
ii) Comprehensive assessment—assessment of suicide risk through static and dynamic factors. Exploration of suicidal intent based on the comprehensive chronological assessment of suicide events (CASE). The CASE approach examines the presenting suicide event (suicidal feelings, ideation, and intent), recent events (over the last 48 h), recent suicide attempts (2 days to 2 months previously), and past suicide attempts (more than 2 months previously) [27]
iii) Formulation of suicide risk—based on a prevention-oriented approach and considers the person’s demographics, culture, history of violence, deliberate self-harm, mental illness, crisis and previous suicide behaviour, current situation (stress, precipitating circumstances), their current risk status (how they compare with a stated population), and risk state (how they compare with themselves at their baseline), their available supports and foreseeable events in their life in order to develop an individualised care plan [28].
iv) Safety planning—performed in collaboration with the consumer, prior to leaving the ED, and includes counselling on access to lethal means, provision of brief patient and carer information, and timely referral for face-to-face mental health follow-up [29, 30].
v) Structured follow-up—within 24–48 h of discharge, in the community [31].
vi) Transition of care plan—“warm handover” is a robust handover to ongoing care services, whether medical/clinical or non-government organisation.

#### Criteria for discontinuing or modifying allocated interventions {11b}

Consumers are made aware (both verbally and written) that they can withdraw consent to participate in the study at any time without any questions asked, or any impact on their ongoing treatment and care. Participants who withdraw from the study are provided with the option of (a) all their collected data being withdrawn from the study or (b) their collected data being used.

#### Strategies to improve adherence to interventions {11c}

Both interventions are delivered in person with each participant. Engagement with the process is noted as part of the clinical intervention and addressed as part of the therapy to facilitate engagement if there are any concerns. Participant clinical progress is discussed in multidisciplinary team (MDT) meetings. Clinical progress and missed sessions are discussed, and strategies for engaging or re-engaging participants are deliberated. This occurs with the recognition of both ASSIP and Brief CBT as standardised, manualised interventions.

The therapy clinician further informs the participant of the therapy processes and expectations. ASSIP and Brief CBT are offered as interventions that are very tightly integrated into the clinical SPP in order to maximise continuity of care and provide the consumer with an integrated care experience, enhancing adherence to interventions. Aspects of the consumer experience and perception of therapy and therapeutic alliance are assessed using the revised Helping Alliance Questionnaire (HAqll).

#### Relevant concomitant care permitted or prohibited during the trial {11d}

Participants can continue to take medications or receive other psychosocial interventions during the study.

#### Provisions for post-trial care {30}

Gold Coast University Hospital and Bond University (the administering institution) will provide insurance cover for the research project (viz., public liability insurance and professional indemnity). Should this study find evidence of the effectiveness of ASSIP or CBT (in addition to TAU) over another treatment arm, consumers will be able to access the effective intervention.

### Outcomes {44}

#### Primary outcome measures

Primary outcomes are the time to representation to hospital with a suicide attempt and proportion representing within 7, 14, 30, and 90 days. Suicidal ideation will also be examined (in a descriptive analysis) to ascertain whether a rise in suicide ideation is commensurate with a fall in suicide attempts (i.e. an increase in help-seeking behaviours). Death by suicide rates will also be examined to make sure that representations with a suicide attempt are not due to participants dying but due to them getting better. Death clearly assessed as not involving self-harm will be represented as not completing the study. Time to suicide attempt is measured by calculating the total number of days from the initial presentation to the re-presenting date for a subsequent suicide attempt. For participants without a subsequent suicide attempt, the total number of days from enrolment to the last assessment (24 months) will be calculated.

#### Secondary outcome measures

Self-reported levels of suicidality, depression, anxiety, stress, resilience, problem-solving skills, and self- and therapist-reported level of therapeutic engagement are measured. Scores will be compared for each group at each follow-up time point. A cost comparison will also be done for both interventions (compared to TAU), and estimates will be based on service information regularly collated by GCMHSS and ED (e.g. contact hours, bed stays, ED visits). We expect a low completion rate (see the “Sample size” section). Study completion will be analysed post hoc, as a secondary outcome.

### Participant timeline {13}

**Fig. 1:**
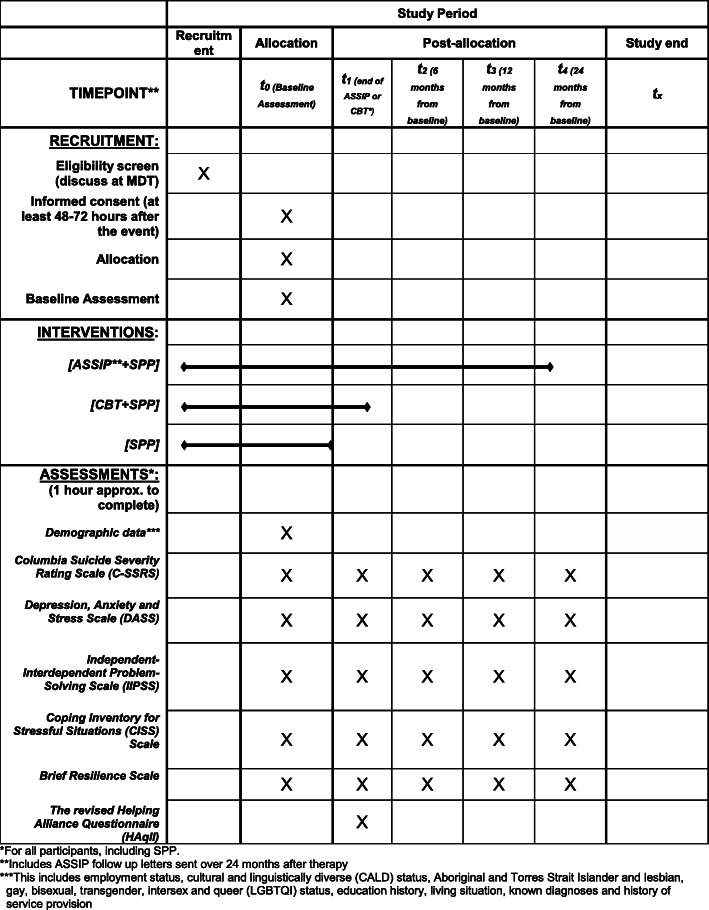
SPIRIT figure—ASSIP-CBT study schedule of recruitment, interventions, and baseline and follow-up assessments. *For all participants, including SPP. **Includes ASSIP follow-up letters sent over 24 months after therapy. ***This includes employment status; cultural and linguistically diverse (CALD) status; Aboriginal and Torres Strait Islander and lesbian, gay, bisexual, transgender, intersex, and queer (LGBTQI) status; education history; living situation; known diagnoses; and history of service provision

Please refer to Fig 1 for the ASSIP-CBT study schedule of recruitment, interventions, and baseline and follow-up assessments (SPIRIT figure).

### Sample size {14}

The sample size was calculated on the basis of a time to event analysis of the time to representation for suicidality. From previous studies using CBT as an additional treatment component following attempted suicide [24, 39], we anticipated that the proportion of patients re-presenting for attempted suicide over a 2-year follow-up period will be approximately half that of the TAU group. From recent data from our health service, this represents a decrease from 48 to 24%. To detect this difference (equivalent to a hazard ratio of 0.42) by the log rank test in a proportional hazards time-to-event analysis with 80% power at a two-sided significance level of 5%, we require 132 participants in two groups of 66 each. However, we anticipated a dropout rate of approximately 4.5% per month which would result in 35% of participants completing the full 24-month follow-up (65% total dropout). The effective dropout rate is thus 41%, as those dropping out later contribute proportionately more. When accounting for this dropout rate, to maintain the same power, a total of 137 participants in each of the three cohorts are required for a total of 411 in total.

### Recruitment {15}

People who present to ED following a suicide attempt (i.e. the act to take their life) are placed on a journey board, which is reviewed by the MDT. The RA regularly monitors journey boards and communicates with clinical teams to recruit potential participants. Any person identified as a potential participant is discussed during the MDT for appropriateness of recruitment to the ASSIP-CBT Research clinic. Names are then provided to the RA who makes contact with the person (obtain formal consent and recruit into the study). The RA will make every reasonable attempt to follow-up eligible participants to recruit into the study.

The characteristics of patients who were eligible for the trial (both participants and non-participants) are also recorded (e.g. age, gender, reason for declining if applicable).

### Assignment of interventions: allocation

#### Sequence generation {16a}

Randomisation occurs after recruitment. Participants are randomised to either SPP, SPP + ASSIP, or SPP + CBT using random block randomisation (ralloc, Stata 15) with blocks of size 6–15. An intention-to-treat approach will be used for analysis.

#### Concealment mechanism {16b}

Allocation is not concealed, and envelopes with participant numbers and containing the group allocation (treatment arm) are opened by the RA (in front of the participants) after formal consent is obtained. Both the RA and the participant do not have prior knowledge of the group allocation details.

#### Implementation {16c}

The RA is responsible for the recruitment of participants and for the assignment of participants to intervention arms. The RA enables the treatment pathway by booking an appointment for the appropriate intervention and informing the ASSIP-CBT Clinician (adult > 25 or youth clinician 16–25, as applicable). The allocation sequence is generated by a biostatistician who is not involved in the recruitment or assessment of participants. Clinical processes and interventions, consumer assessment/documentation, and treatments as well as follow-up, are not affected by the recruitment to the ASSIP-CBT study and are progressed by treating teams as clinically appropriate, as per usual practices.

### Assignment of interventions: blinding

#### Who will be blinded {17a}

Given the clear differences in the therapy interventions, participants and those administering the interventions are not blinded. However, those assessing the outcomes will be blinded to group assignment.

#### Procedure for unblinding if needed {17b}

Not applicable, as those responsible for the healthcare of the participant are not blinded to the group assignment and can inform the statistician to remove data related to a participant with a particular participant number.

### Data collection and management

#### Plans for assessment and collection of outcomes {18a}

Each person who presents to Gold Coast Health is assigned a unique identifying, or UR, number. The Emergency Information System at Gold Coast Health (viz., FirstNet database) records all ED presentations. The ED presentations will be interrogated for the UR number of all study participants, and any presentation within 24 months of an initial presentation will be reviewed for evidence of suicidality. Multivariate Cox proportional hazard regression models will be used to analyse time to re-presentation for a suicide attempt. Time to suicide attempt is measured by calculating the total number of days from the initial presentation (not including days of admission for some consumers with medical and/or psychiatric admission) to the re-presenting date for a subsequent suicide attempt.

For secondary outcomes, data are collected by the RA at five time points: (1) baseline, (2) end of CBT or ASSIP interventions, (3) 6 months, (4) 12 months, and (5) 24 months from baseline. At baseline, demographic data and psychometric data are collected in person or via the telephone with the RA, as outlined below. At end of treatment and 6, 12, and 24 months, psychometric data are collected. Therapeutic alliance is assessed at the completion of the interventions. Cost comparisons will be based on service information regularly collated by GCMHSS and ED (e.g. contact hours, bed stays, ED visits). Study completion will be analysed post hoc, as a secondary outcome.

i) Demographic data: includes age, gender, method of suicide attempt, employment status, education history, place of residence, living situation, marriage status, family contact, known diagnoses and co-occurring conditions (e.g. drug and alcohol use), and history of service provision, including use of psychological interventions

It is anticipated that a number of people within the study population will be from specific cultural groups, who may also be high-risk populations, such as Aboriginal and Torres Strait Islander people, people from cultural and linguistically diverse (CALD) communities, people who identify as LGBTQI, older persons (aged over 60 years), and young people (aged 16–18 years). Questions are included to enable the identification of these specific target population subgroups.

ii) Psychometric data: participants are assessed on their level of suicidality, depression, anxiety, stress, resilience, therapeutic engagement, and their ability to cope and problem solve. The following measures are used.

Columbia Suicide Severity Rating Scale (C-SSRS) [44] is a rating scale that assesses suicide ideation and behaviour. The scale consists of four subscales: severity of ideation (e.g. plan or method), intensity of ideation (e.g. frequency, duration), behaviour (e.g. attempts, preparatory behaviour, non-suicidal self-harm), and lethality (for suicide attempts). The C-SSRS has been used in clinical and non-clinical populations, including adolescents and adults [44], and has been validated in other countries including Korea [45] and Turkey [46]. The C-SSRS has demonstrated convergent and divergent validity with other established suicide assessment scales (i.e. Scale for Suicide Ideation and the Columbia Suicide History Form) [44] and has also shown to have high sensitivity and specificity for suicidal behaviour compared to the other scales [44]. The intensity of the ideation subscale has also demonstrated good internal consistency [44]. The C-SSRS was originally developed by the US Food and Drug Administration to be used in clinical research trials and is currently recommended as the preferred instrument for clinical trials [47]. The C-SSRS takes around 10 min to complete.

Depression, Anxiety and Stress Scale (DASS)-21 [48–50] is the short form of DASS, a self-report scale that aims to measure depression (e.g. dysphoria, hopelessness), anxiety (e.g. situational anxiety, subjective experience of anxious affect), and stress (e.g. difficulty relaxing, feeling nervous, being easily upset). Each subscale (i.e. depression, anxiety, and stress) contains 7 4-point (0–3) Likert scale items. A final score for each subscale is obtained by summation with higher scores indicating higher levels of depression, anxiety, or stress. Scores are multiplied by 2 (to be consistent with scores from the full version of DASS) to give the final score which can range from 0 to 42. The DASS has been used and established in clinical and non-clinical populations and has been shown to have high internal consistency and convergent and divergent validity [48, 51, 52]. The scale takes around 5–10 min to complete.

The Coping Inventory for Stressful Situations (CISS) [53–55] is a 48-item self-report questionnaire that assesses thee different types of coping styles: emotional-orientated, task orientated, and avoidant (distraction and social diversion):

- Task-oriented coping: primary control style—the main emphasis is to solve the problem or alter the situation while controlling emotions. This can be helpful when situations are changeable but not helpful for complex social problems.
- Emotion-oriented coping: secondary control style—the main emphasis is to reduce emotional stress and emotional reactions. Can be helpful in the short-term for situations that are uncontrollable but will be maladaptive in the long term.
- Avoidant-distracted coping: focuses on pleasurable or distracting activities that helps one avoid the problem or situation. Can be helpful in the short term but will not be helpful over the long term especially for problems that are uncontrollable.
- Avoidant-social coping: focuses on social diversion as a means of distracting oneself from the problem or situation (or seeking assistance to address the problem or situation). Can be helpful in the short term, but it is more effective to learn how to address problems directly without continually relying on others.

The CISS has shown high internal consistency for clinical and non-clinical samples [55]. The CISS has also demonstrated moderate to high test-retest reliability [55]. Construct validity has been demonstrated through factor analysis work and relationships between the CISS and other measures [55]. The CISS includes separate adolescent and adult forms [55]. Participants are asked to rate each item on a 5-point frequency scale: 1 “not at all” to 5 “very much”. The CISS usually takes around 10 min to complete.

Brief Resilience Scale (BRS) is a 6-item outcome measure designed to assess the ability to bounce back or recover from stress [56]. The BRS is considered to relate more closely to the original meaning of resilience [56]. The BRS has been shown to be a reliable and valid measure of resilience in clinical and non-clinical samples [56, 57], and in a systematic review of resilience measurement scales, the BRS was deemed to have one of the best psychometric ratings [58]. The scale takes around 5 min to complete.

The revised Helping Alliance Questionnaire (HAqII) is a 19-item self-report questionnaire (therapist and patient version) which is an improved version of the 11-item self-rating Penn Helping Alliance Questionnaire (HAq) [59]. It is used to evaluate the quality of the patient–therapist relationship (therapeutic alliance). The HAqII has demonstrated good validity for psychotherapy outcomes, good internal consistency for both patient and therapist (Cronbach’s alpha 0.9–0.93), and good test-retest reliability (test-retest coefficient patient 0.78; therapist 0.56) [59]. The HAqII demonstrated high convergence with another, widely used self-report measure of alliance California Psychotherapy Alliance Scale (CALPAS) total score (patient version: *r* = 0.59–0.69 and therapist version: *r* = 0.75–0.79) [59]. The questionnaire takes around 10 min to complete.

Independent-Interdependent Problem-Solving Scale (IIPSS): the IIPSS assesses problem-solving preference: independent problem-solving (e.g. problem-solving without relying on others’ assistance) and interdependent problem-solving (problem-solving relying on others’ assistance). The scale has good reliability, with a single factor structure (eigenvalue = 3.96) and good internal consistency (Cronbach’s *α* = .77 and .80) [60]. The scale also has good convergent validity (relationship with Relational-Interdependent Self-Construal Scale and Extraversion Scale) and good predictive validity (predicting student’s likelihood of either finding a solution online or asking another student for help) [60].

#### Plans to promote participant retention and complete follow-up {18b}

The RA contacts participants via telephone call (or other contact means such as emails or text messages) to complete the later stages of the follow-up questionnaires. The RA also monitors the retention rate and reasons for discontinuation of the study (e.g. consent withdrawn, lost to follow-up).

#### Data management {19}

All assessment data are entered electronically into a database (Microsoft Excel) and scanned into the Consumer Integrated Mental Health Application (CIMHA). Any publication of study results involves de-identified data only, and all study data and information are stored according to established research protocols (paper questionnaires digitised and then securely destroyed, video data stored on secure Queensland Health servers with restricted access), with research team members being supervised by experienced investigators. Participant video files are maintained in storage for a period of 5 years after completion of the study.

#### Confidentiality {27}

All participants are assigned a participation number when they consent to join the study to ensure information pertaining to their treatment and health outcomes is de-identified during analysis and reporting. All medical/mental health records kept become part of the participants’ medical records and are protected by Gold Coast Health policy (for the State of Queensland, Australia) regarding information privacy and disclosure. This is only viewable by treating clinicians accessing the participants’ medical records and is planned for routine use in clinical studies in Gold Coast Health

#### Plans for collection, laboratory evaluation, and storage of biological specimens for genetic or molecular analysis in this trial/future use {33}

This is not applicable, as no biological samples are collected.

## Statistical methods

### Statistical methods for primary and secondary outcomes {20a}

To determine the effectiveness of additional CBT + SPP or ASSIP+SPP compared with SPP alone, univariate (log rank test) and multivariate Cox proportional hazard shared frailty regression models and multilevel mixed effects parametric models will be used to analyse time to re-presentation for suicide attempts [35]. Time to suicide attempt is measured by calculating the total number of days from the initial presentation (with days of psychiatric admission for some consumers taken into account), to the re-presenting date for a subsequent suicide attempt and may include multiple representations [35]. For participants without a subsequent suicide attempt, the total number of days from enrolment to the last assessment (24 months) will be calculated. All collected demographic and clinical variables will be considered in the model-building process. Variables with *P* ≤ 0.1 on univariate analysis will be included in all combinations and retained in the model if *P* < 0.05. Plausible interactions of retained variables will be tested. Collinearity of potential model variables will be assessed using the variance inflation factor. Where appropriate, the proportional hazards assumption will be tested using Schoenfeld residuals. Kaplan-Meier plots will allow visual comparisons between the groups. Analyses will be undertaken on an intention to treat basis. Stata 15 (Stata Corp. College Station, TX, USA) will be used for statistical analyses.

### Interim analyses {21b}

Not applicable; no interim analyses will be conducted for this study.

### Methods for additional analyses (e.g. subgroup analyses) {20b}

Not applicable as no subgroup analyses are planned though significant interaction effects will be used to suggest a differential effect of treatment across different patient groups.

### Methods in analysis to handle protocol non-adherence and any statistical methods to handle missing data {20c}

All participants will be included in the main analysis (intention to treat) regardless of dropout or adherence to the intervention. Only those who withdraw from the study and all their collected data will be excluded from the analysis. Missing data on the secondary outcomes will be handled by multiple imputation if missingness is found to be “completely at random” and > 10%.

### Plans to give access to the full protocol, participant level-data, and statistical code {31c}

We plan to make de-identified aggregate data available, in keeping with relevant privacy policy and considerations and subject to a formal agreement with the requesting institution.

### Oversight and monitoring

#### Composition of the coordinating centre and trial steering committee {5d}

##### Steering committee (CIs based at GCUH)

Study design and conception, preparation of protocol and revisions, organising working group meetings, reporting to clinical governance, recruitment, data collection, and dissemination of findings.

##### International collaborators (subgroup)

Expertise assessment training (CIs KM, AP), ASSIP training, data analysis, and dissemination of findings.

##### ASSIP-CBT Working Group (subgroup)

Study planning, referral pathways, advice for lead investigators, quality assurance, treatment as usual pathway, and clinical intervention procedures.

##### Research Clinical Supervision Group and MDT meetings (subgroups)

Training, supervision, all subgroups report to the steering committee. This study was formally monitored by the Gold Coast Health Office for Research Governance and Development - Clinical trials monitoring officer (see section 23).

#### Composition of the data monitoring committee, its role, and reporting structure {21a}

No Data Safety Monitoring Board has been appointed for this study. The study is considered low risk by HREC, and care is as per current Gold Coast Health guidelines for consumers, which already follows a leading postvention model (the SPP). The ASSIP-CBT steering committee provides oversight on the ongoing conduct, safety, and progress of the trial.

#### Adverse event reporting and harms {22}

All adverse events (reported and observed) will be reported to HREC, and recorded by the research team, with respect to any of the following types of harm occurring in the study, and the likelihood, severity, and consequence of those harms occurring.

##### Physical harm

People recruited into the study have by inclusion criteria made a suicide attempt and have thus physically harmed themselves. There is at baseline further risk of self-harm for many of these people, although the study interventions aim to prevent further attempts at self-harm, and the clinical pathway that participants will engage in employs constant monitoring for suicidality and risk of self-harm.

##### Psychological harm

All the interventions employed have an evidence base to demonstrate a reduction in psychological harm and distress and do not cause such distress. One area that was identified by the Lived Experience Suicide Prevention Research Advisory Committee that required careful disclosure to participants is the randomisation process, which may result in people not receiving a novel therapy (CBT or ASSIP) but rather receiving treatment as usual (i.e. SPP).

Under the guidance of our Gold Coast Health Consumer Representative and our Gold Coast Health Carer Representative, there has already, and will continue to be substantial interface with the lived experience and consumer community. This will aid to gauge the participants’ and communities’ expectations of the research, and we are being guided by the Committee to manage any relevant limitations and risks of the research project. The Lived Experience Suicide Prevention Research Advisory Committee assisted in drafting particular wording into the participant information and consent form (PICF) to explain this study to participants in understandable language. The Lived Experience Committee also wrote the first section of the PICF as a letter from themselves to the prospective participant, as a way to engage and connect with prospective participants via shared lived experience. This was strongly endorsed by the HREC.

CBT has independent evidence of its general acceptance among consumers with lived experience of suicide and suicidal behaviour and ASSIP has been developed from hundreds of narrative interviews with patients who had attempted suicide, and the key characteristic of ASSIP is the patient-centred therapeutic approach (including the video-playback in the second session). The Lived Experience Committee was given detail on the CBT and ASSIP interventions, perceived the therapies as being positive and endorsed their use.

Some of the questions in the questionnaire ask about topics which participants might find sensitive or which may bring back upsetting memories associated with their suicide attempt (e.g. C-SSRS, ASSIP interviews). The Lived Experience Suicide Prevention Research Advisory Committee has advised for the RA to give a brief overview of the questionnaire before starting (i.e. an overview of the types of questions being asked and how long the process will take). The Lived Experience Committee also reviewed all the questions in the questionnaire and made a number of suggestions around appropriate language use (i.e. making sure that the language use is recovery orientated). Minor changes were made to some of the languages (e.g. “wish to be dead” changed to “have you wished you were not alive anymore”; “medical damage” changed to “physical harm”). The Lived Experience Committee also advised on the order in which the different psychometric measures would be given to a participant to optimise the participant experience, for example, attempting the resilience scale which focuses on positive attributes last.

Participants are supported by someone when they fill in the questionnaires, and additional help is made available should they experience any distress as a result of completing the questionnaires. Participation in this study is voluntary, and participants are made aware that they can withdraw their participation at any time without any impact on current ongoing treatment and care.

If the consumer’s risk variables (e.g. suicide risk) increase during the intervention, or during a research activity session, than standard procedures of crisis service referral applies (e.g. Acute Care Team, GP), clinicians and the RAs also ensure that participants are aware of who to contact in crisis (e.g. after-hours crisis services such as the Mental Health hotline).

#### Frequency and plans for auditing trial conduct {23}

The Office for Research Governance and Development has a risk-based monitoring programme to enhance the safety of participants and staff involved in clinical research trials. This monitoring programme also provides overall research oversight and mitigates potential risks to the participants and the institution. Research trials conducted at Gold Coast Health are screened and reviewed to determine on-site monitoring frequency and intensity. This process is independent from the investigators and research team.

A data-monitoring officer, who reports to the Gold Coast Health Research Governance Office, has been appointed to the study trial. The data-monitoring officer ensures research integrity of the trial, including reviewing research procedures (e.g. informed consent and safety reporting procedures), assessing data management procedures, and evaluating risks of the current research monitoring plan.

#### Plans for communicating important protocol amendments to relevant parties (e.g. trial participants, ethical committees) {25}

Any protocol amendments (e.g. change of study objectives, study design, recruitment procedures, questionnaire changes) will be submitted to HREC for approval. The funding body (Suicide Prevention Australia) will also be notified of any modifications. If current participants are affected by the amendments, they will be notified, and additional consent will be requested. Online trial registries will also be updated on the changes, if applicable. Any minor changes to the protocol (i.e. changes that do not affect how the study is being conducted) will be agreed upon by the main steering committee and CIs of the trial, if applicable.

#### Dissemination plans {31a}

Findings will be communicated via relevant GCMHSS committees and stakeholders, as well as published in peer-reviewed journals. The findings will also be presented at relevant national and international conferences. Participants can also obtain a summary of the findings by including their email in the participant information and consent form or at their request.

## Discussion

This paper describes the study protocol for the randomised control trial that assesses whether adding one of two psychological interventions (ASSIP or Brief CBT for Suicide Prevention) to a standard clinical care approach (SPP) will improve the outcomes for consumers presenting to a public health service with a suicide attempt. Currently, there are limited treatment options, with limited underpinning research, for those who present to ED with suicidal behaviour. The primary outcomes of this study are re-presentation to hospitals with suicide attempts. Suicidal ideation will also be examined to see whether a rise in suicide ideation is commensurate with a fall in suicide attempts (potentially indicating an increase in help-seeking behaviours). Self-reported levels of suicidality, depression, anxiety, stress, resilience, problem-solving skills, and self- and therapist-reported level of therapeutic engagement are also being examined.

### Strengths

This research study will move both ASSIP and Brief CBT from efficacy to effectiveness research. This study is outcome-focused, with clear aims of assessing the effect on suicidal representation following the addition of each of two different structured psychological interventions to treatment as usual. A cost comparison of the interventions compared to TAU is also planned, thus delivering evidence related to practical implementation as well as clinical efficacy and effectiveness. We emphasise the pragmatic approach of examining two interventions which are sustainable in a large and busy service, where clinicians are trained in these interventions, allowing for rapid potential translation into standard clinical practice. This study is also future-oriented, as it is aligned with national and international strategic priorities, recognises the continued growth of South-East Queensland, and emphasises a systems approach (i.e. the coordination and integration of existing services at state and national level) to suicide prevention [13]. Finally, this innovative work has the design features that will enable its future translation to other services with similar frameworks in Queensland and elsewhere.

### Limitations

A potential limitation of this study is the exclusion of people with an established diagnosis of borderline personality disorder; this exclusion may limit the generalisability of the findings and prevent a real-world appraisal of cost-effectiveness. As the study is being conducted in a “real-world” clinical setting, the inclusion/exclusion study has to take into account clinical pragmatism as well as the potential for harm during psychological treatments. People with borderline personality disorder traits or vulnerability can still be recruited into the study at the clinician’s discretion following MDT discussion. However, people with an established diagnosis with moderate to severe borderline personality disorder will not be recruited to the study. The main concern with recruiting consumers with established diagnoses of borderline personality disorders is the risk of re-traumatising, as they share their story during the ASSIP intervention. As participants are randomly assigned, we cannot control for allocation to the ASSIP arm of the study. During the ASSIP first session, the individual provides a narrative of what led to the suicide attempt; at this stage, the therapist reduces any prompting and lets the person self-reflect and share as far back to their history as they wish. This can be counterproductive for those with complex trauma and risk emotion dysregulation in the individual [61]. Therefore, ASSIP is not recommended for consumers with a history of complex trauma as this may trigger dissociative states (K Michel 2018, developer of ASSIP).

Another potential limitation could be the therapist’s allegiance to a particular type of therapy or therapy model [18]. There may also be differences in therapists’ skills and competencies between the two therapies. Clinicians in this study are delivering both the ASSIP and the Brief CBT for Suicide Prevention (and have received training for both). The steps we have taken to reduce this potential bias include providing opportunities for supervision (that directly addresses any therapy adherence or allegiance issues) and the application of an ASSIP therapy adherence scale [62]. In addition, both models are manualised, so this should mitigate any therapist biases. Finally, it is possible that participants may seek to represent in some other setting, and therefore, we will not capture them in our data records. Examples are people who may present interstate (our service is located close to a state border) or who might present to a primary healthcare provider. The study is being conducted in a “real world” setting; as such, we follow a pragmatic approach in terms of what can and cannot be controlled for.

### Operational issues

There were some initial issues in establishing the appropriate clinical referral pathway for study recruitment. There were also some staff concerns about the impact of the study on business as usual, as well as some confusion around the recruiting and consenting processes. Clinical referral pathways have now been established and embedded into our usual business practice. The RA also frequently rounds each of the hospital wards and mental health community settings to assist clinicians with referral and recruitment. The RA obtains formal consent from participants. The embedding of these processes will allow for rapid translation of these evidence-based interventions for suicide into our standard clinical practice.

There were some initial delays in the recruitment of research staff due to the process of establishing contracts for staff; staff were recruited to GCMHSS, but their wages were being paid by the administrating institution (university) where the grant funds are being managed. Staff could not be directly recruited to the administrating institution (as initially planned) as they would not be able to access the clinical records or data contained in the clinical information systems used within the GCMHSS. The process of requesting external access to Queensland health clinical data and records could also only be granted after approval has been obtained by Public Health Act (PHA). By recruiting research staff directly to GCMHSS, the staff were able to have immediate access to clinical data and records and thus preventing delay in study recruitment. The GCMHSS invoices the administrating institution for the staff wages.

## Conclusion

In Australia, suicide, in addition to the personal loss of life and the mental and emotional impact and burden on those close to people who suicide, is also associated with national economic costs estimated at $17.5 billion annually [63]. This cost has been calculated based on service and prevention programme costs, lost productivity among survivors, and years of life lost [63]. Responding to, and providing care to, people presenting to hospitals and health services with suicide risk is identified as a priority for action by the Queensland Suicide Prevention Health Taskforce [13]. Data from Queensland Health found that almost 25% of those who died by suicide had previously made contact with a health service within 7 days prior to their death [13]. This finding highlights the pivotal role that hospital emergency departments play in the engagement and assessment of those at acute risk of suicide.

The study is conducted in busy clinical settings thus testing the applicability of these interventions for public mental health services and to consumers from different cultural backgrounds, ages, genders, and presenting diagnoses. This study hopes to provide evidence for a suicide prevention, assessment, and intervention package, available for immediate clinical translation for use in Australian settings. Moreover, we hope to bridge the gap between service users and researchers by including people with lived experience of suicide as active contributors to this research study.

## Trial status

This manuscript refers to study protocol version 1, 5 April 2019. The recruitment for this study started in October 2019. We have recruited 12 people so far. The interventions have also commenced. We are currently at the beginning stages of year 2. Due to the COVID-19 pandemic, we had to pause the trial for 2 months. We also added an amendment to HREC to allow us to recruit via telephone/online methods. Recruitment has now recommenced. Recruitment is expected to be completed at the end of 2022.

## Abbreviations

GCUH: Gold coast university hospital
CBT: Cognitive behavioural therapy
ASSIP: Attempted suicide short intervention program
SPP: Suicide prevention pathway
GCMHSS: Gold coast mental health and specialist services
HREC: Human research ethics committee (gold coast)
CIMHA: Consumer integrated mental health application
C-SSRS: Columbia suicide severity rating scale
DASS-21: Depression, anxiety and stress scale 21-item
CISS: The coping inventory for stressful situations
BRS: Brief resilience scale
IIPSS: Independent-interdependent problem-solving scale
